# Estimation of measurement uncertainty for the quantification of protein by ID-LC–MS/MS

**DOI:** 10.1007/s00216-023-04705-8

**Published:** 2023-05-26

**Authors:** Ashley Beasley-Green, N. Alan Heckert

**Affiliations:** 1 Material Measurement Laboratory (Biomolecular Measurement Division), National Institute of Standards and Technology, 100 Bureau Drive, Gaithersburg, MD 20899-8390 USA; 2 Information Technology Laboratory (Statistical Engineering Division), National Institute of Standards and Technology, 100 Bureau Drive, Gaithersburg, MD 20899-8390 USA

**Keywords:** Measurement uncertainty, Isotope-dilution liquid chromatography-tandem mass spectrometry (ID-LC–MS/MS), Albumin, Design of experiments (DOE), Reference measurement procedure (RMP), Standard Reference Material® (SRM)

## Abstract

The emergence of mass spectrometry (MS)-based methods to quantify proteins for clinical applications has led to the need for accurate and consistent measurements. To meet the clinical needs of MS-based protein results, it is important that the results are traceable to higher-order standards and methods and have defined uncertainty values. Therefore, we outline a comprehensive approach for the estimation of measurement uncertainty of a MS-based procedure for the quantification of a protein biomarker. Using a *bottom-up* approach, which is the model outlined in the “Guide to the Expression of Uncertainty of Measurement” (GUM), we evaluated the uncertainty components of a MS-based measurement procedure for a protein biomarker in a complex matrix. The cause-and-effect diagram of the procedure is used to identify each uncertainty component, and statistical equations are derived to determine the overall combined uncertainty. Evaluation of the uncertainty components not only enables the calculation of the measurement uncertainty but can also be used to determine if the procedure needs improvement. To demonstrate the use of the *bottom-up* approach, the overall combined uncertainty is estimated for the National Institute of Standards and Technology (NIST) candidate reference measurement procedure for albumin in human urine. The results of the uncertainty approach are applied to the determination of uncertainty for the certified value for albumin in candidate NIST Standard Reference Material® (SRM) 3666. This study provides a framework for measurement uncertainty estimation of a MS-based protein procedure by identifying the uncertainty components of the procedure to derive the overall combined uncertainty.

**Graphical Abstract:**

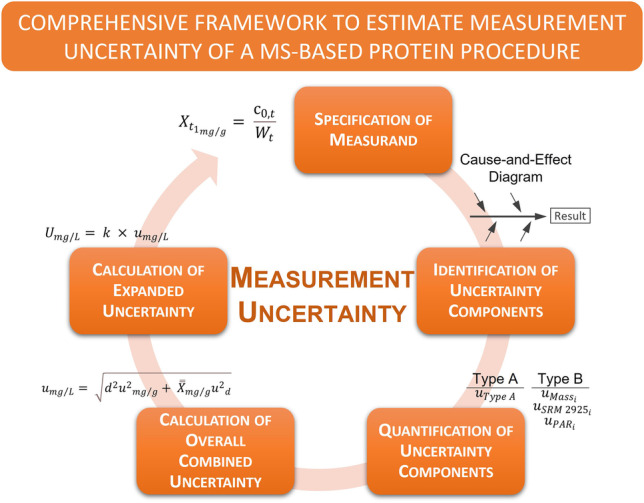

**Supplementary Information:**

The online version contains supplementary material available at 10.1007/s00216-023-04705-8.

## Introduction

Mass spectrometry (MS)–based quantitative proteomics has emerged as an important tool used in clinical laboratories for the evaluation of clinical protein biomarkers for disease diagnosis and management. Therefore, the accuracy and comparability of these MS-based protein results are essential for healthcare practitioners to provide precise and consistent clinical decisions for patient care. To anchor the clinical utility of MS-based protein measurements, it is of utmost importance that protein results are traceable to higher-order standards and methods and have defined uncertainty values. Measurement uncertainty as defined in the International Vocabulary of Metrology (VIM) is a “*parameter, associated with the result of a measurement, that characterizes the dispersion of the values that could reasonably be attributed to the measurand*” [1]. Estimating the measurement uncertainty of MS-based protein results is essential to understand the impact of uncertainty on (1) the measurement results and (2) the suitability of the results for clinical decisions. The objective of this study is to provide a comprehensive assessment of measurement uncertainty for a procedure that applies isotope dilution-liquid chromatography-tandem MS (ID-LC–MS/MS) for the quantification of a protein biomarker in a clinical matrix. This is the first paper to outline, in detail, the individual uncertainty components that contribute to the overall combined uncertainty of a MS-based measurement procedure for protein quantification. The “Guide to the Expression of Uncertainty of Measurement” (GUM) [2], which establishes the guidelines for evaluating and expressing measurement uncertainty, is applied to identify and quantify the individual uncertainty sources of a MS-based protein procedure that contribute to the overall combined uncertainty. The model proposed in the GUM is termed, in application, the *bottom-up* approach [3, 4]. In this study, we evaluate the measurement uncertainty of the NIST candidate reference measurement procedure (RMP), which incorporates ID-LC–MS/MS for the quantification of albumin in human urine [5]. The NIST candidate RMP is based on the detection and measurement of signature proteotypic (typically trypsin) peptides that uniquely and stoichiometrically represent albumin [5]. An intact, full-length isotopically labeled (^15^N-labeled) recombinant albumin protein is incorporated in the measurement procedure as an internal standard (IS) for the absolute quantification of albumin in urine [5]. The procedure couples ID-LC–MS/MS with the multiple reaction monitoring (MRM) MS scan mode to selectively target signature tryptic albumin peptides. The ratio of unlabeled analyte to ^15^N-labeled IS is used to generate a calibration curve for the determination of albumin in a urine sample. In accordance with the *bottom-up* approach [2–4], a cause-and-effect diagram of the NIST candidate RMP is used to identify the sources of uncertainty and statistical models are applied to estimate the overall combined uncertainty. The results of the measurement uncertainty assessment are used to determine the overall combined uncertainty of the certified value for albumin content in candidate NIST Standard Reference Material® (SRM) 3666, which will be used to establish metrological traceability for routine clinical results of albumin in urine and enhance the accuracy and confidence of clinical decisions for kidney disease.

This study provides a framework for the comprehensive assessment of the individual uncertainty components that contribute to the measurement uncertainty of a MS-based protein quantitative procedure.

## Materials and methods

### Chemicals

NIST SRM 2925 Recombinant Human Serum Albumin Solution (Primary Reference Calibrator for Urine Albumin) (Frozen), candidate SRM 3666 Albumin and Creatinine in Frozen Human Urine, full-length ^15^N-labeled recombinant human serum albumin (*r*HSA) IS (Albumin Biosciences; Huntsville, AL), 99% label incorporation determined via LC–MS/MS analysis (see Electronic Supplementary Material, Table S1 and Fig. S1), Trypsin-Gold MS-grade (Promega, Madison, WI, USA), Dithiothreitol (DTT, Pierce), Iodoacetamide (IAM, Pierce), high-purity LC–MS-grade water/0.1% (*volume fraction*), formic acid and acetonitrile (ACN)/0.1% (*volume fraction*) formic acid (Honeywell Burdick and Jackson).

### Sample preparation

**Fig. 1:**
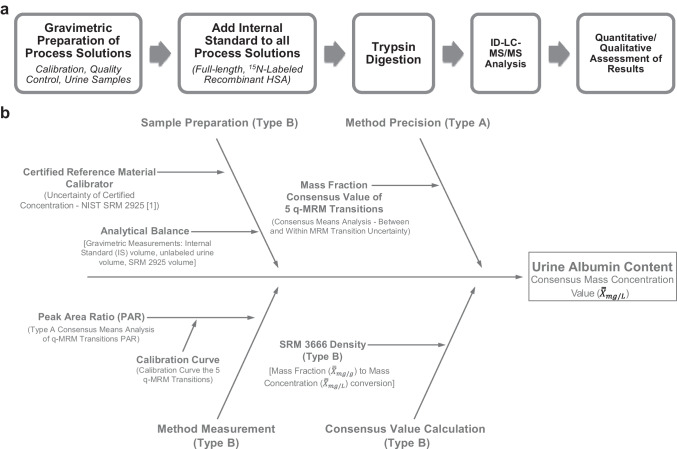
Detailed protocol for NIST candidate RMP [5] (**a**) (see “Methods” section for more details) and the cause-and-effect diagram of measurement procedure (**b**)

Preparation of the calibration solutions, quality control material, and candidate SRM 3666 samples is outlined in detail in ref. [5] and illustrated in Fig. 1a. To prepare the calibration and quality control (QC) solutions, stock and working solutions are gravimetrically prepared using SRM 2925 (unlabeled analyte) and an isotopically labeled IS (^15^N-labeled recombinant HSA). Multiple vials of candidate SRM 3666 (level 1 to level 4) are randomly selected from the material lot. Prior to trypsin digestion, the IS is added to each process sample and each analysis set consists of calibrants, QCs, and candidate SRM 3666 process samples for each level. The process samples are allowed to solubilize overnight at 4°C prior to trypsin digestion.

### Protein digestion

The enzymatic (trypsin) protein digestion protocol is outlined in ref. [5]. Each analysis set (calibrants, QCs, and candidate SRM 3666 samples) is incubated at 97°C for 10 min to denature albumin and cooled to room temperature (RT; ≈25°C). The samples are reduced with 5 mmol/L dithiothreitol at 60°C for 30 min, followed by alkylation with 15 mmol/L iodoacetamide at RT for 30 min in the dark. An approximate 1:30 mass ratio of trypsin-to-total protein is used for digestion, and the digestion reaction is conducted at 37°C for 24 h. Following digestion, the pH of the samples is reduced with 50 mL/L formic acid in water and incubated for 45 min at 37°C to quench the digestion reaction. The samples are concentrated (no heat) overnight and resuspended in 100 µL of 0.1% (*volume fraction*) formic acid in water the next day for tandem MS analysis.

### LC–MS/MS analysis

Analysis of the digested samples is performed on an Agilent 6460 triple quadrupole mass spectrometer in positive ion mode equipped with an Agilent 1290 Series LC system utilizing an Agilent Zorbax 300 SB-C18 column (2.1 mm × 150 mm, 3.5 μm). The column temperature is maintained at 45°C, and the peptides were loaded onto the column at a flow rate of 200 μL/min in 97% (*volume fraction*) mobile phase A (water with 1 mL/L formic acid) and 3% (*volume fraction*) mobile phase B (ACN with 1 mL/L formic acid). General mass spectrometric conditions: gas temperature of 300°C; gas flow of 7 L/min; nebulizer of 20 psi (1.4 × 10^5^ Pa); sheath gas temperature of 300°C; sheath gas flow of 6 L/min; capillary voltage of 4000 V; and a nozzle voltage of 1500 V. Analysis of the process samples is conducted in a randomized sequence with replicate measurements to reduce influence of systematic bias on the output measurements.

### Quantitative method

Integration of chromatographic peaks for both the unlabeled albumin and IS is performed using the Agilent MassHunter Quantitative Analysis software (Version B.10.00). All peak integrations were manually confirmed and corrected, as needed. A total of 46 measurements (11 peptides with 2 or 3 MRM transitions per peptide) are collected for the 23 MRM transitions in each sample. Peak areas from MassHunter integration are imported to Microsoft Excel for manual quantitative assessment of the raw data. The peak area and concentration ratios of the calibration solutions are used to generate a linear calibration curve for each transition and the albumin content of the quality control and candidate SRM 3666 samples is determined from the calibration curves.

### Design of experiments (DOE) assessment

**Fig. 2:**
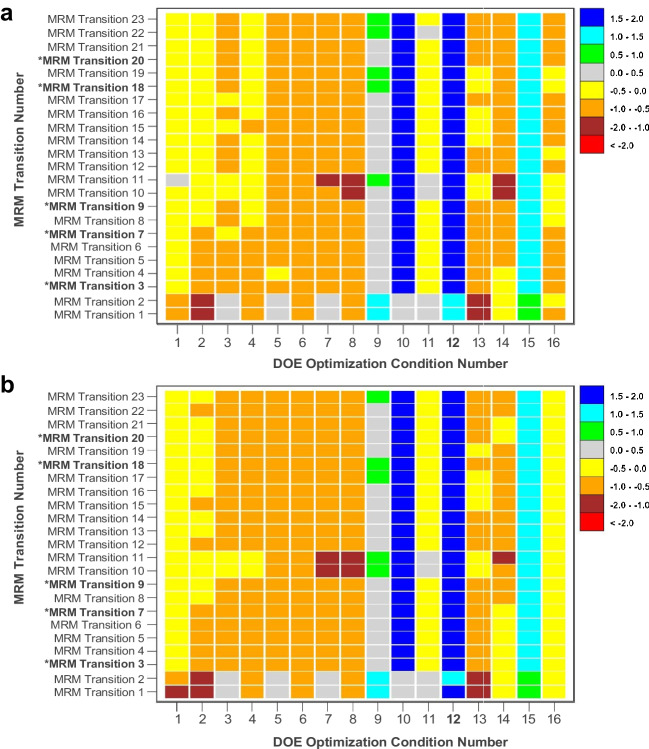
*Z*-score map of the DOE optimization study results (*n* = 8) for 23 MRM transitions (unlabeled SRM 2925 (**a**) and ^15^N-labeled IS (**b**)) for trypsin digestion process in NIST candidate RMP [5] for quantification of albumin in urine (*denotes the *qt*-MRM transitions)

The DOE optimization study is applied to statistically determine the optimal trypsin digestion conditions for albumin, to reduce the uncertainty of the candidate RMP. In addition to reducing the uncertainty, the study is used to minimize the number of MRM transitions used for quantitative assessment of albumin in urine. The central composite design (CCD) of the DOE optimization study is composed of a full-factorial matrix with the following parameters: a three-factor design (2^3^; *X*_1_, trypsin-to-protein ratio; *X*_2_, digestion reaction time; *X*_3_, digestion reaction temperature), five (5) levels per factor, two (2) center points with six (6) replicates, and six (6) star points (± *α*) (see Experimental Supplemental Material, Table S2). The value for *α* in the three-factor design is 1.684, using *α* = [2^* k*^]^1/4^, where *k* is 3 for the number of factors. The raw peak area measurements of the MRM transitions from the DOE optimization study are used to generate the z-score maps for SRM 2925 (unlabeled) and the IS (^15^N-labeled) (Fig. 2).

### Measurement uncertainty

Estimation of measurement uncertainty is performed in accordance with the GUM [2]. Of the six steps involved in calculating measurement uncertainty, the following steps are applied: specification of the measurand; identification of uncertainty components; quantifying uncertainty; calculating the combined uncertainty; and calculating the expanded uncertainty. The measurand is expressed as a mathematical equation based on the experimental measurement procedure and the cause-and-effect diagram is used to identify the uncertainty sources. The uncertainties of individual components are evaluated and quantified using analytical data. The NIST DATAPLOT [6, 7] software, a public-domain statistical analysis software package, is used for the consensus means analysis and calculation of the type A uncertainty (DSL-HHD and DSL-bootstrap).

## Results and discussion

The NIST candidate RMP, a MS-based quantitative procedure for albumin in urine, is used to illustrate how the *bottom-up* approach [2–4] can be applied for the determination of overall combined uncertainty for a MS-based measurement procedure.

### Specification of measurand

A total of 23 MRM transitions (11 MRM peptides with 2 to 3 MRM transitions/peptide) are used in the NIST candidate RMP for albumin quantification in urine [5] (Table 1). The measurand for the candidate RMP is the consensus mass concentration (${\overline{\overline{X}}}_{\mathrm{mg}/\mathrm{L}}$) value of albumin in urine determined by combining replicate measurements of each MRM transition. Calculation of ${\overline{\overline{X}}}_{\mathrm{mg}/\mathrm{L}}$ is a multistep process, with the first step being the determination of the mass fraction (${X}_{\mathrm{mg}/\mathrm{g}}$) for each MRM transition. The following equation is used to calculate the albumin mass fraction (${X}_{\mathrm{mg}/\mathrm{g}}$) result for a given MRM transition (*t*_*1*_-MRM transition, ${X}_{{{t}_{1}}_{\mathrm{mg}/\mathrm{g}}}$):

1 $${X}_{{{t}_{1}}_{\mathrm{mg}/\mathrm{g}}}= \frac{{\mathrm{c}}_{0,t}}{{W}_{t}}$$

where ${c}_{0,t}$ is the *t*_*1*_*-*MRM transition concentration ratio (mass of urine sample to mass of ^15^N-labeled IS) derived from the linear calibration curve of *t*_*1*_*-*MRM transition. ${\mathrm{W}}_{t}$ is the mass fraction of the IS to urine material mass. The density and concentration of the IS are used for mass unit conversion. The ${\mathrm{W}}_{t}$ component in Eq. 1 is derived from:

2 $${\mathrm{W}}_{t}= \frac{\frac{{m}_{\mathrm{IS}} }{{d}_{IS}}\times {C}_{\mathrm{IS}}}{{\mathrm{m}}_{U}} \times 1000,$$

where ${m}_{\mathrm{IS}}$ is the mass of the IS, ${d}_{\mathrm{IS}}$ is the density of the IS, *C*_IS_ is the mass concentration of the IS, and ${m}_{U}$ is the mass of the unknown urine sample. Because the concentration ratio (${\mathrm{c}}_{0,t}$) is derived from the calibration curve of *t*_*1*_***-***MRM transition, ${X}_{{{t}_{1}}_{\mathrm{mg}/\mathrm{g}}}$ (mass fraction) is also given as:

3 $${X}_{{{t}_{1}}_{\mathrm{mg}/\mathrm{g}}}=\frac{{PAR}_{t}- {B}_{0,t}}{{\mathrm{B}}_{1,t}}\times \frac{1}{{W}_{t}},$$

where ${PAR}_{t}$ is the *t*_*1*_***-***MRM transition peak area ratio (unlabeled *t*_*1*_***-***MRM transition in urine sample to IS *t*_*1*_***-***MRM transition, ^15^N-labeled IS) and ${B}_{0,t}$ and ${B}_{1,t}$ represent the *y*-intercept and the slope of the linear calibration curve for T1, respectively. The consensus mass fraction value (${\overline{\overline{X}}}_{\mathrm{mg}/\mathrm{g}}$) is calculated by combining the ${X}_{t,\mathrm{mg}/\mathrm{g}}$ values for the MRM transitions using consensus mean analysis via the DerSimonian-Laird (DSL) model (random-effects model) [8, 9]:

4 $$\begin{array}{l}{y}_{ij}= \mu + {m}_{i}+ {\varepsilon }_{ij}\\ i=1, 2\dots , {n}_{mm};j=\mathrm{1,2},\dots ,{n}_{i}\end{array}$$

where $i$ indexes the MRM peptide, $j$ indexes the number of replicates within a given MRM peptide, ${n}_{mm}$ represents the number of MRM peptides, ${n}_{i}$ represents the number of replicates within a given MRM peptide, $\mu$ represents the grand mean for all MRM peptides/transitions, ${m}_{i}$ represents the mean of given MRM peptide (relative to grand mean), and ${\varepsilon }_{ij}$ represents variance between the MRM peptides. The final step in the determination of albumin in urine is the calculation of the consensus mass concentration (${\overline{\overline{X}}}_{\mathrm{mg}/\mathrm{L}}$) from ${\overline{\overline{X}}}_{\mathrm{mg}/\mathrm{g}}$ and the density of the urine material (${d}_{\mathrm{urine}}\times 1000$):

5 $${\overline{\overline{X}}}_{\mathrm{mg}/\mathrm{L}}={\overline{\overline{X}}}_{\mathrm{mg}/\mathrm{g}}\times \left( {d}_{\mathrm{urine}}\times 1000\right)$$

**Table 1:** MRM transition list for quantification of albumin in urine using NIST candidate RMP [5]

Peptide type—*Qt* or *Qa*^a^	MRM transition number	Peptide	Precursor *m/z*	Product *m/z*	IS precursor *m/z*	IS product *m/z*
Qt	**Transition 3**	**TYETTLEK**	**492.75**	**720.30**	**497.23**	**727.40**
Qt	**Transition 7**	**VFDEFKPLVEEPQNLIK**	**682.37**	**900.00**	**689.35**	**909.50**
Qt	**Transition 9**	**FQNALLVR**	**480.78**	**685.40**	**487.27**	**695.40**
Qt	**Transition 18**	**LCTVATLR**	**467.26**	**660.40**	**472.75**	**669.40**
Qt	**Transition 20**	**YLYEIAR**	**464.25**	**651.30**	**469.24**	**659.30**
Qa	Transition 1	DLGEENFK	476.22	723.3	481.21	731.30
Qa	Transition 2	DLGEENFK	476.22	229.07	481.21	231.07
Qa	Transition 4	TYETTLEK	492.75	265.10	497.23	279.20
Qa	Transition 5	VFDEFKPLVEEPQNLIK	682.37	970.50	689.35	981.50
Qa	Transition 6	VFDEFKPLVEEPQNLIK	682.37	712.40	689.35	721.37
Qa	Transition 8	FQNALLVR	480.78	276.09	487.27	279.09
Qa	Transition 10	QTALVELVK	500.81	488.27	506.29	493.25
Qa	Transition 11	QTALVELVK	500.81	587.30	506.29	593.40
Qa	Transition 12	RPCFSALEVDETYVPK	637.65	961.50	644.30	972.40
Qa	Transition 13	RPCFSALEVDETYVPK	637.65	244.17	644.30	247.16
Qa	Transition 14	LVAASQAALGL	507.30	189.08	513.29	191.08
Qa	Transition 15	LVAASQAALGL	507.30	712.40	513.29	721.40
Qa	Transition 16	LVNEVTEFAK	575.31	937.40	581.29	947.40
Qa	Transition 17	LVNEVTEFAK	575.31	694.40	581.29	701.40
Qa	Transition 19	LCTVATLR	467.26	274.12	472.75	276.12
Qa	Transition 21	YLYEIAR	464.25	277.2	469.24	279.10
Qa	Transition 22	AEFAEVSK	440.72	201.05	445.21	203.04
Qa	Transition 23	AEFAEVSK	440.72	680.32	445.21	687.30

*m/z* mass-to-charge ratio, *IS* internal standard

^a^The “Peptide type” differentiates the quantitative (Qt) and qualitative (Qa) MRM transitions and the five Qt-MRM transitions are in bold

### Identification of uncertainty components

The individual uncertainty components relevant to the candidate RMP are depicted in the cause-and-effect diagram in Fig. 1b. The uncertainty components are divided into four categories: sample preparation, method precision, method measurement, and consensus value calculation. The uncertainty associated with the concentration value of the certified reference material (NIST SRM 2925), the unlabeled calibrant, is obtained from the certificate of analysis [10, 11].

### Quantification of uncertainty

The uncertainty evaluation is based on the information derived from the cause-and-effect diagram (Fig. 1b), and the four sources of uncertainty are categorized into two types: type A and type B. The type A uncertainty component is represented by the method precision component. Type A uncertainty is established by statistical analysis of the albumin mass fraction values (${X}_{\mathrm{mg}/\mathrm{g}}$) of the MRM transitions and is represented by the within and between MRM peptide variance [1, 12–15]. The DSL random-effects model (Eq. 4), in conjunction with either the Horn-Horn-Duncan (HHD) variance method [15] or the parametric bootstrap variance method [16–22], is applied to determine the type A uncertainty (${u}_{\mathrm{Type A}}$) for ${\overline{\overline{X}}}_{\mathrm{mg}/\mathrm{g}}$ [6, 7]. The more conservative uncertainty value of the two methods (DSL-HHD or DSL-bootstrap) is selected as the ${\overline{\overline{X}}}_{\mathrm{mg}/\mathrm{g}}$ type A uncertainty.

Type B uncertainty is established by non-statistical analysis of the factors associated with calculation of the urine albumin consensus values: ${\overline{\overline{X}}}_{\mathrm{mg}/\mathrm{g}}$ and ${\overline{\overline{X}}}_{\mathrm{mg}/\mathrm{L}}$ [1, 12–15]. The type B uncertainty sources that contribute to the ${\overline{\overline{X}}}_{\mathrm{mg}/\mathrm{g}}$ combined uncertainty *(*${u}_{\mathrm{mg}/\mathrm{g}}$) are sample preparation, method measurement, and consensus value calculation (Fig. 1b). The sample preparation source represents the uncertainty of the certified concentration value of NIST SRM 2925 (${u}_{\mathrm{SRM }2925}$) and the uncertainty associated with the analytical balance (${u}_{\mathrm{Mass}}$) for the gravimetric preparations of the calibration, QC, and urine samples. SRM 2925 is the unlabeled material used, in conjunction with the labeled IS, to generate the linear calibration curve and there is a 1.1% contribution from ${u}_{\mathrm{SRM }2925}$ to ${u}_{\mathrm{mg}/\mathrm{g}}$ [10, 11]. The gravimetric measurements using an analytical balance (${u}_{\mathrm{Mass}}$) represents 0.1% contribution to ${u}_{\mathrm{mg}/\mathrm{g}}$ (Fig. 1b). The method measurement component represents uncertainty of PAR (${u}_{\mathrm{PAR}}$) for each MRM peptide, which is the ratio of the raw peak area output of the unlabeled (calibrant solution or urine sample) to the ^15^N-labeled IS, and the uncertainty for the linear calibration curve (${u}_{\mathrm{ccur}}$). As shown in Eq. 3, PAR is used to generate the linear calibration curve; therefore, ${u}_{\mathrm{ccur}}$ is combined with ${u}_{\mathrm{PAR}}$. Uncertainty of PAR (${u}_{\mathrm{PAR}}$) is established by consensus means analysis of the PAR results for the MRM transitions (DSL-HHD or DSL-bootstrap), which is the same method used to determine ${u}_{\mathrm{Type A}}$ for ${\overline{\overline{X}}}_{\mathrm{mg}/\mathrm{g}}$ (Eq. 4). As performed in the ${u}_{\mathrm{Type A}}$ estimate for ${\overline{\overline{X}}}_{\mathrm{mg}/\mathrm{g}}$, the more conservative uncertainty value of the DSL-HHD [15] and DSL-bootstrap [22] methods is selected as the type A uncertainty for PAR (${u}_{\mathrm{PAR},\mathrm{Type A}}$). PAR is a key component of the procedure because it is used to construct the calibration curve for each MRM transition, which is used to determine the albumin content of the unknown sample (Eq. 3). The dependence of the calibration curve on PAR supports incorporating ${u}_{\mathrm{ccur}}$ with ${u}_{\mathrm{PAR}}$ (total uncertainty of PAR).

Variability in PAR can impact both the precision of the calibration curve and the accuracy of ${\overline{\overline{X}}}_{\mathrm{mg}/\mathrm{g}}$ and, ultimately, ${\overline{\overline{X}}}_{\mathrm{mg}/\mathrm{L}}$. Therefore, the digestion procedure should be optimized to enhance the quality of the injected material, to improve the precision of the instrument output measurements (raw peak area), and to reduce the impact of PAR (${u}_{\mathrm{PAR}}$) on the overall combined uncertainty. The DOE optimization approach is applied to determine the optimal enzymatic digestion conditions of the measurement procedure (Fig. 2) [5]. A detailed description of the DOE optimization approach is outlined in the Electronic supplemental material. By applying the DOE optimization approach, we are able to access a wide range of digestion conditions in a single experiment. The output data (raw peak area) for the DOE optimization study for each MRM transition of both the unlabeled (SRM 2925) and labeled IS material are used to generate a *z*-score map (Fig. 2). As illustrated in Fig. 2, the highest *z*-score, or highest peak area output value, across the 23 MRM transitions for both unlabeled and labeled IS material are observed in Digestion Condition #12 (Fig. 2), which represents the following parameter settings: enzyme-to-protein mass ratio of 1:30; digestion reaction time of 23 h; and digestion reaction temperature of 37.0°C. In addition to determining the optimal digestion condition, using the data from the DOE optimization approach, we are also able to determine the optimal MRM transitions for quantification of albumin in urine. By selecting the MRM transitions with consistently high peak area measurements, the total number of MRM transitions used to quantify albumin is reduced from 23 to 5. The 23 MRM transitions are divided into two groups: quantitative (*qt-*) and qualitative (*qa-*) MRM transitions. The consensus values (${\overline{\overline{X}}}_{\mathrm{mg}/\mathrm{g}}$ and ${\overline{\overline{X}}}_{\mathrm{mg}/\mathrm{L}}$) and associated uncertainties (${u}_{\mathrm{mg}/\mathrm{g}}$ and ${u}_{\mathrm{mg}/\mathrm{L}}$) represent the combined results of the 5 *qt*-MRM transitions (Table 1). Optimizing the digestion conditions and reducing the *qt*-MRM transitions decrease the measurement uncertainty by improving the precision of the peak area results and decreasing the number of measurements used to calculate the consensus values (${\overline{\overline{X}}}_{\mathrm{mg}/\mathrm{g}}$ and ${\overline{\overline{X}}}_{\mathrm{mg}/\mathrm{L}}$) and uncertainties (${u}_{\mathrm{mg}/\mathrm{g}}$ and ${u}_{\mathrm{mg}/\mathrm{L}}$).

### Calculation of overall combined uncertainty

The uncertainty components associated with the NIST candidate RMP are combined to derive ${\overline{\overline{X}}}_{\mathrm{mg}/\mathrm{g}}$ combined uncertainty (${u}_{\mathrm{mg}/\mathrm{g}}$) using:

6 $${u}_{\mathrm{mg}/\mathrm{g}}= \sqrt{{{u}^{2}}_{\mathrm{Type\; A}} +{{u}^{2}}_{\mathrm{Type \;B}} },$$

where ${u}_{\mathrm{Type A}}$ represents the type A uncertainty of ${\overline{\overline{X}}}_{\mathrm{mg}/\mathrm{g}}$ determined via the DSL-HHD or DSL-bootstrap methods and the ${u}_{\mathrm{Type B}}$ represents the combined type B uncertainties [6, 7]. The ${u}_{\mathrm{type B}}$ component is determined from the quantified uncertainty sources (${u}_{\mathrm{SRM }2925}$, ${u}_{\mathrm{Mass}}$, ${u}_{\mathrm{PAR}}$) using the following:

7 $${u}_{\mathrm{Type B}}= \sqrt{{{u}^{2}}_{{\mathrm{Mass}}_{i}}+{{u}^{2}}_{{\mathrm{SRM }2925}_{i}}+{{u}^{2}}_{{\mathrm{PAR}}_{i}}},$$

The ${u}_{\mathrm{PAR}}$ represents the combined uncertainty for ${u}_{\mathrm{PAR},\mathrm{Type A}}$ and ${\overline{u} }_{\mathrm{ccur}}$ for the 5 *qt*-MRM transitions:

8 $${u}_{\mathrm{PAR}}= \sqrt{{{u}^{2}}_{\mathrm{PAR},\mathrm{Type \;A}}+{{\overline{u} }^{2}}_{\mathrm{ccur}}},$$

where ${\overline{u} }_{\mathrm{ccur}}$ represents the mean calibration curve uncertainty (${\overline{u} }_{\mathrm{ccur}}$) of the 5 *qt*-MRM transitions. The ${u}_{\mathrm{ccur}}$ value for each of the five (5) *qt*-MRM transitions is calculated using [23–25]:

9 $${u}_{\mathrm{ccur}}=\frac{{S}_{x/y}}{{B}_{1}}\sqrt{\frac{1}{k}+\frac{1}{n}+ \frac{{\left({y}_{j}- {\overline{y} }_{i}\right)}^{2}}{{{B}_{1}}^{2} \sum {\left({x}_{i}- {\overline{x} }_{i}\right)}^{2}}},$$

where ${B}_{1}$ is the slope of the linear equation (Eq. 3), $k$ represents the number of replicate measurements of the unknown urine sample, $n$ represents the total number of data points on calibration curve, ${x}_{i}$ is the individual *x*-value (*x*-axis) on the calibration curve, ${\overline{x} }_{i}$ represents the mean value of the ${x}_{i}$ values, and ${\overline{y} }_{i}$ represents the mean value of the ${y}_{i}$ values (PAR values). The value represents the PAR value of the analyte (albumin) measured in the unknown urine sample. The ${S}_{x/y}$ value represents the standard error of the linear calibration curve, which is derived from [23–25]:

10 $${S}_{x/y}=\sqrt{ \frac{\sum {\left({y}_{i}- \widehat{y}\right)}^{2}}{n-2}}.$$

To determine the ${\overline{\overline{X}}}_{\mathrm{mg}/\mathrm{L}}$ combined uncertainty (${u}_{\mathrm{mg}/\mathrm{L}}$), the density of the urine material (${d}_{\mathrm{urine}}$) and the density uncertainty (${u}_{\mathrm{d}}$) are combined with ${\overline{\overline{X}}}_{\mathrm{mg}/\mathrm{g}}$ and ${u}_{\mathrm{mg}/\mathrm{g}}$. The final combined standard uncertainty (${u}_{\mathrm{mg}/\mathrm{L}}$) of the consensus mass concentration value (${\overline{\overline{X}}}_{\mathrm{mg}/\mathrm{L}}$) for the 5 *qt*-MRM transitions is calculated using:

11 $${u}_{\mathrm{mg}/\mathrm{L}}=\sqrt{{d}^{2}{{u}^{2}}_{\mathrm{mg}/\mathrm{g}}+ {\overline{\overline{X}}}_{\mathrm{mg}/\mathrm{g}}{{u}^{2}}_{\mathrm{d}}},$$

### Calculating the expanded uncertainty

The ${u}_{\mathrm{mg}/\mathrm{g}}$ and ${u}_{\mathrm{mg}/\mathrm{L}}$ values are expressed as expanded uncertainties ($U$), which are obtained by multiplying the combined standard uncertainty (${u}_{\mathrm{mg}/\mathrm{g}}$ or ${u}_{\mathrm{mg}/\mathrm{L}}$) by a coverage factor ($k$) [1, 12–15]:

12 $${U}_{\mathrm{mg}/\mathrm{g}}= k \times {u}_{\mathrm{mg}/\mathrm{g}}\mathrm{ or }{U}_{\mathrm{mg}/\mathrm{L}}= k \times {u}_{\mathrm{mg}/\mathrm{L}},$$

where $k=2$ is used for calculation of the expanded uncertainty (approximately 95% confidence level) of the NIST candidate RMP.

### Application of measurement uncertainty evaluation

The NIST candidate RMP [5] is used to certify the mass fraction and mass concentration content of albumin in candidate SRM 3666. SRM 3666 is a four-level material composed of pooled human urine with endogenous albumin levels within the clinical ranges for urine albumin (normal: 0 to 30 mg/L; microalbumin: 30 to 150 mg/L; microalbumin: ≥ 150 mg/L) [26]. Using the NIST candidate RMP, the consensus values (${\overline{\overline{X}}}_{\mathrm{mg}/\mathrm{g}}$, ${\overline{\overline{X}}}_{\mathrm{mg}/\mathrm{L}}$) are determined for each level of candidate SRM 3666 (level 1 to level 4). The overall combined standard uncertainty (${u}_{\mathrm{mg}/\mathrm{g}}$ and ${u}_{\mathrm{mg}/\mathrm{L}}$) for each level is determined using Eq. 6 (${u}_{\mathrm{mg}/\mathrm{g}}$) and Eq. 10 (${u}_{\mathrm{mg}/\mathrm{L}}$) and the associated expanded uncertainty (${U}_{\mathrm{mg}/\mathrm{g}}$ and ${U}_{mg/L}$) for each level is determined using Eq. 12. The concentration of endogenous albumin in candidate SRM 3666 (level 1 to level 4) with the associated expanded uncertainties (*k* = 2) are level 1—8.28 mg/L ± 1.12 mg/L, level 2—31.11 mg/L ± 2.64 mg/L, level 3—112.77 mg/L ± 10.78 mg/L, and level 4–360.50 mg/L ± 31.11 mg/L, so that 13.53% (L1), 8.49% (L2), 9.56% (L3), and 8.63% (L4) of the respective concentration gave an estimated expanded uncertainty. The ${\overline{\overline{X}}}_{\mathrm{mg}/\mathrm{g}}$ and ${\overline{\overline{X}}}_{\mathrm{mg}/\mathrm{L}}$ values with the associated measurement uncertainties, in detail, for candidate SRM 3666 (level 1 to level 4) are shown in Tables 2 and 3, respectively.

**Table 2:** Combined uncertainty results for urine albumin consensus mass fraction values (${\overline{\overline{X}}}_{\mathrm{mg}/\mathrm{g}}$) for level 1 to level 4 of candidate NIST SRM 3666

SRM 3666 level number	Urine albumin mass fraction consensus value (${\overline{\overline{X}}}_{\mathrm{mg}/\mathrm{g}}$)	Type A uncertainty (mg/g) (${u}_{\mathrm{Type A}}$)	Mass-analytical balance uncertainty (${u}_{\mathrm{Mass}}$)	SRM 2925 concentration value uncertainty (${u}_{\mathrm{SRM} 2925}$)	PAR Uncertainty (${u}_{\mathrm{PAR}}$)	Standard combined uncertainty (${u}_{\mathrm{mg}/\mathrm{g}}$) of urine albumin mass fraction consensus value	Expanded combined uncertainty (${U}_{\mathrm{mg}/\mathrm{g}}$) of urine albumin mass fraction consensus value
Level 1	0.00816	0.00027	0.00001	0.00009	0.00047	0.00055	0.00110
Level 2	0.03068	0.00062	0.00003	0.00035	0.00109	0.00130	0.00260
Level 3	0.11079	0.00314	0.00011	0.00126	0.00407	0.00529	0.01059
Level 4	0.35500	0.00242	0.00036	0.00404	0.01457	0.01532	0.03064

**Table 3:** Combined uncertainty results for urine albumin consensus mass concentration values (${\overline{\overline{X}}}_{\mathrm{mg}/\mathrm{L}}$) for level 1 to level 4 of candidate NIST SRM 3666

SRM 3666 level number	Urine albumin mass fraction consensus value (${\overline{\overline{X}}}_{\mathrm{mg}/\mathrm{g}}$)	Standard combined uncertainty (${u}_{\mathrm{mg}/\mathrm{g}}$) of urine albumin mass fraction consensus value	SRM 3666 density value (g/mL)	SRM 3666 density value standard uncertainty (g/mL)	Urine albumin mass concentration consensus value (${\overline{\overline{X}}}_{\mathrm{mg}/\mathrm{L}}$)	Standard combined uncertainty (${u}_{\mathrm{mg}/\mathrm{L}}$)—urine albumin mass concentration consensus value	Expanded combined uncertainty (${U}_{\mathrm{mg}/\mathrm{L}}$)—urine albumin mass concentration consensus value
Level 1	0.00816	0.00055	1015.19	0.00029	8.28395	0.55870	1.11739
Level 2	0.03068	0.00130	1013.97	0.00024	31.10860	1.31985	2.63970
Level 3	0.11079	0.00529	1017.84	0.00029	112.76649	5.38884	10.77770
Level 4	0.35500	0.01532	1015.49	0.00035	360.49895	15.55610	31.11230

**Fig. 3:**
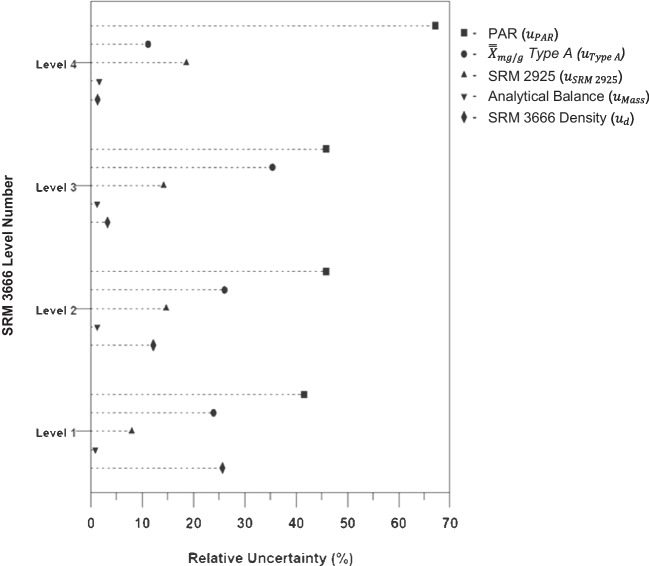
Contribution of the different uncertainty sources to the overall combined uncertainty of the consensus mass concentration value (${u}_{\mathrm{mg}/\mathrm{L}}$) of albumin in urine using the NIST candidate RMP [5]. The method factors and associated uncertainty components: PAR—${u}_{\mathrm{PAR}}$, type A—${u}_{\mathrm{Type A}}$, Certified material (NIST SRM 2925)—${u}_{\mathrm{SRM }2925}$, Analytical balance—${u}_{\mathrm{Mass}}$, and density of NIST SRM 3666—${u}_{\mathrm{d}}$)

Figure 3 illustrates the relative contribution of the uncertainty components on the overall combined standard uncertainty (${u}_{\mathrm{mg}/\mathrm{g}}$ and ${u}_{\mathrm{mg}/\mathrm{L}}$) for the four levels of candidate SRM 3666. The percent contribution of the ${\overline{\overline{X}}}_{\mathrm{mg}/\mathrm{g}}$ combined uncertainty (${u}_{\mathrm{mg}/\mathrm{g}}$) across the four levels revealed that type B uncertainty is considerably higher than type A uncertainty using the NIST candidate RMP. The influence of the analytical balance source (${u}_{\mathrm{Mass}}$) on ${u}_{\mathrm{mg}/\mathrm{g}}$ is insignificant, as shown by the low relative uncertainty for ${u}_{\mathrm{Mass}}$. According to Fig. 3, of the four uncertainty components (sample preparation, method measurement, method precision and consensus value calculation), the method precision (${u}_{\mathrm{PAR}}$) represents a major source of uncertainty for the measurement procedure. This suggests that the calibration curve (${\overline{u} }_{\mathrm{ccur}}$ included in ${u}_{\mathrm{PAR}}$ calculation), instrument precision, and protein digestion have a significant contribution on the measurement uncertainty of a MS-based protein quantification procedure. The impact of PAR on the combined uncertainty (${u}_{\mathrm{mg}/\mathrm{g}}$) estimate supports the use of the DOE optimization approach to reduce the measurement uncertainty by establishing the optimal enzymatic digestion conditions and by condensing the number of *qt*-MRM transitions used to calculate ${\overline{\overline{X}}}_{\mathrm{mg}/\mathrm{g}}$ and ${\overline{\overline{X}}}_{\mathrm{mg}/\mathrm{L}}$.

## Conclusion

The use of MS-based methods for clinical measurements of protein biomarkers has heightened the need for accurate and comparable results for healthcare practitioners to provide precise and consistent patient care. Estimation of the measurement uncertainty of MS-based clinical procedures is essential to better understand the influence of the procedure parameters on the final measurement result and to, ultimately, determine the clinical suitability of the clinical result. Therefore, in this study, we provide a comprehensive assessment of measurement uncertainty of a MS-based measurement procedure for the quantification of a protein biomarker in a clinical matrix. To our knowledge, this is the first study that applies the *bottom-up* approach [3, 4], which is the model outlined in the GUM [2], to identify and quantify the individual uncertainty components that contribute to the combined uncertainty for a measurement procedure that incorporates ID-LC–MS/MS for the absolute quantification of a protein biomarker. The *bottom-up* approach is applied to determine the combined uncertainty for the NIST candidate RMP used for the determination of albumin in human urine [5]. The cause-and-effect diagram is used to identify the uncertainty components of the candidate RMP, and mathematical equations are derived to quantify the contribution of each component toward the combined uncertainty of the procedure. The results of the measurement uncertainty assessment for the candidate RMP are applied to certify albumin in NIST candidate SRM 3666, which is intended as a matrix-based (human urine) certified reference material to validate the accuracy of routine methods used in clinical laboratories and to, ultimately, establish metrological traceability of clinical urine albumin results to the International System of Units (SI). In summary, this study provides a framework for the estimation of measurement uncertainty for a MS-based procedure validated for the purpose of quantifying protein.

## Supplementary Information

Below is the link to the electronic supplementary material.

Supplementary file1 (PDF 630 KB)

## Declarations

### Disclaimer

Certain commercial equipment, instruments, and materials are identified in this paper in order to specify the experimental procedure adequately. Such identification is not intended to imply recommendation or endorsement by NIST, nor is it intended imply that the materials or equipment are necessarily the best available for the purpose.
