# Supplementary material for: Estimation of measurement uncertainty for the quantification of protein by ID-LC–MS/MS
Source: Anal Bioanal Chem. 2023 May 26;415(16):3265–74. doi: 10.1007/s00216-023-04705-8 (PMC10287794; doi:10.1007/s00216-023-04705-8)

# SUPPLEMENTAL INFORMATION

# ESTIMATION OF MEASUREMENT UNCERTAINTY FOR THE QUANTIFICATION OF PROTEIN BY ID-LC-MS/MS

**Ashley Beasley-Green**[1] and Alan Heckert[2]

[1]Material Measurement Laboratory (Biomolecular Measurement Division), [2]Information

Technology Laboratory (Statistical Engineering Division)

National Institute of Standards and Technology, Gaithersburg, MD

**Journal Name:**
Analytical and Bioanalytical Chemistry

**Corresponding Author Information:**
**Ashley Beasley-Green, Ph.D.**
National Institute of Standards and Technology
100 Bureau Drive, MS 8314
Gaithersburg, Maryland 20899-8390
Email: ashley.beasley@nist.gov

## A. INTERNAL STANDARD $^{15}$N-LABEL INCORPORATION ASSESSMENT

To determine the degree of isotope ($^{15}$N) enrichment of the $^{15}$N-labeled full-length recombinant human serum albumin (HSA) internal standard (IS), the isotopic distribution of twelve (12) tryptic albumin peptides via LC-MS/MS. The experimental isotopic distribution of the twelve (12) tryptic peptides, including the 11 peptides used for the NIST candidate RMP [5], was compared to the theoretical isotope distribution to determine the degree of isotope enrichment. The NIST Isotope Enrichment Calculator program (*https://www.nist.gov/services-resources/software/isotope-enrichment-calculator*) was used to determine the theoretical isotopic distribution and assess the experimental isotopic distribution of the twelve (12) tryptic peptides. The program output was a label incorporation percentage and the Pearson correlation coefficient (*r*) value, which is a measure of linear correlation between the experimental and theoretical isotopic distribution of the selected peptide.

**Experimental Design**. The samples were digested via the trypsin digestion protocol included in the NIST candidate RMP [5] and the digested samples were analyzed via LC-MS/MS using an Orbitrap Elite (Thermo Scientific) ion trap mass spectrometer. The Orbitrap Elite was operated in the positive ion mode using CID. The high resolution (R=120,000) full MS data was processed manually to assess the peak intensities of the isotopic distribution of the twelve (12) tryptic peptides.

**Results/Discussion.** The isotope enrichment of the $^{15}$N-labeled IS was determined using the peak intensity data [three process replicates (n=36)]. The average $^{15}$N incorporation for the IS was 99.7% (CV of 0.5 %) with an average *r*-value of 0.9999 (CV of 0.02 %) (Table S1). Figure S1 illustrates the theoretical and experimental (observed) isotopic distribution for unlabeled (NIST SRM 2925) and $^{15}$N-labeled IS tryptic peptides: YLYEIAR, TYETTLEK and QTALVELVK. The isotopic distribution of the NIST SRM 2925 (unlabeled) is equivalent to that of the 0 % theoretical isotopic probability distribution and the $^{15}$N-Labeled material is equivalent to that of the 99 % theoretical isotopic probability distribution.

**Table S1.** Percent label incorporation (Incor) and r-value for the twelve peptides for the $^{15}$N-labeled IS.

| Peptide | $^{15}$N-Labeled-Sample 1 | | $^{15}$N-Labeled-Sample 2 | | $^{15}$N-Labeled-Sample 3 | |
|---|---|---|---|---|---|---|
| | %Incor | r-value | %Incor | r-value | %Incor | r-value |
| AEFAEVSK | 98.9 | 1.0000 | 98.1 | 1.0000 | 99.4 | 1.0000 |
| DLGEENFK | 100 | 1.0000 | 100.2 | 1.0000 | 99.8 | 1.0000 |
| FQNALLVR | 99.8 | 1.0000 | 100.2 | 1.0000 | 99.9 | 1.0000 |
| LVAASQAALGL | 99.9 | 1.0000 | 100.2 | 1.0000 | 99.6 | 0.9999 |
| LVNEVTEFAK | 98.8 | 1.0000 | 99.2 | 1.0000 | 100.2 | 1.0000 |
| LVTDLTK | 99.5 | 1.0000 | 100.7 | 1.0000 | 99.6 | 1.0000 |
| QTALVELVK | 99.9 | 1.0000 | 99.9 | 1.0000 | 100.7 | 1.0000 |
| RPCFSALEVDETYVPK | 99.8 | 0.9998 | 99.7 | 1.0000 | 99.8 | 0.9998 |
| SLHTLFGDK | 99.5 | 1.0000 | 100 | 1.0000 | 99.5 | 1.0000 |
| TYETTLEK | 99.2 | 1.0000 | 100.2 | 1.0000 | 98.9 | 1.0000 |
| VFDEFKPLVEEPQNLIK | 99.6 | 0.9994 | 99.9 | 0.9990 | 99.9 | 0.9992 |
| YLYEIAR | 99.1 | 1.0000 | 99.4 | 1.0000 | 100.6 | 1.0000 |
| | %Incor | r-value | | | | |
| Average (n=36) | 99.71 | 0.9999 | | | | |
| Standard Deviation | 0.54 | 0.0002 | | | | |
| %CV | 0.5 | 0.0 | | | | |

**Fig S1** Comparison of theoretical isotopic probability distribution versus experimental (observed) isotopic peak distribution from tandem MS (Orbitrap Elite) spectra for tryptic peptides: YLYEIAR, TYETTLEK, and QTALVELVK. The experimental (observed) $^{15}$N-label incorporation percentage for NIST

SRM 2925 (unlabeled, blue) and $^{15}N$-Labeled IS (red) are overlaid with the theoretical $^{15}N$ incorporation percentages of 100 % (black), 99 % (green), 50 % (light blue) and 0 % (gray) for each peptide.

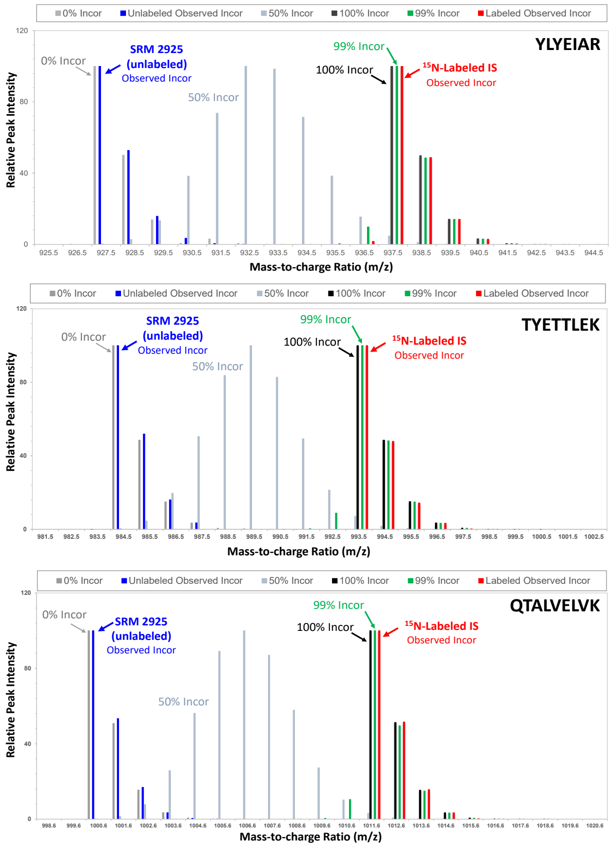


## B. DESIGN OF EXPERIMENT (DOE) OPTIMIZATION STUDY

To reduce the PAR uncertainty ($u_{PAR}$) of the National Institute of Standards and Technology (NIST) candidate reference measurement procedure (RMP) [5], the design of experiment (DOE) Optimization study was conducted to optimize trypsin digestion protocol of the measurement of albumin. The DOE Optimization study was performed to establish the optimal settings for the trypsin digestion protocol of the candidate RMP [5].

**Experimental Design**. The Response Surface Method (RSM) was applied using the Central Composite Design (CCD). The design consisted of the following: full factorial matrix ($2^3$), two center points (red) with six (6) replicates per sample, and six (6) star points (± α) (Figure S2). The α value of 1.684 was determined using the following equation:

$$\alpha = [2^k]^{1/4} = 2^{3/4} \quad \text{Eq. (1)}$$

where k is 3 for the number of factors. The three-factors included in the design were: enzyme (trypsin)-to-protein ratio ($X_1$), digestion reaction time ($X_2$), and digestion reaction temperature ($X_3$). Consistent with the Screening Study, a total of 23 measurements (11 peptides with 2 or 3 MRM transitions per

peptide) were collected for each sample SRM 2925 (unlabeled albumin) and $^{15}$N-labeled recombinant HSA (IS) were collected for each factor-level combination (Total of 16 Conditions). The five levels for each factor are outlined in Table S2 and the parameters for each of the 16 Optimization Conditions are outlined in Table S3. To analyze the data, the peak area results for SRM 2925 and the IS were converted to z-scores.

**Results/Discussion.** All 11 MRM peptides (SRM 2925 and IS) were observed in the eight (8) conditions (Figure S3). The MRM peak area ratio (PAR) values and the performance of the IS for the 16 conditions across the MRM transition are consistent, as illustrated in Figure S4 and S5, respectively. Using the peak area results for both SRM 2925 (unlabeled) and IS to generate the z-score graph for each optimization condition, the optimal condition (highest z-score across all MRM peptides) for trypsin digestion of albumin (unlabeled and IS) was Optimization Sample #12 (enzyme (trypsin)-to-protein mass ratio ($X_1$) of 1:30; digestion reaction time ($X_2$) of 23 h; digestion reaction temperature ($X_3$) of 37.0 °C) (Table S3).

**Conclusion.** The DOE optimization approach was used to statistically determine the optimal trypsin digestion conditions for albumin to achieve the highest response from the NIST LC-MS/MS method. From the data, we observe that the optimal condition (highest z-score across all MRM peptides) for trypsin digestion of albumin (unlabeled and IS) was Optimization Sample #12. By optimizing the trypsin conditions for albumin and identifying the quantitative MRM peptides/transitions, we can determine the content of albumin in human urine samples with the highest degree of confidence and precision. The urine albumin trypsin digestion protocol and LC-MS/MS method are fit-for-purpose to accomplish value-assignment of the candidate NIST SRM 3666 Albumin and Creatinine in Frozen Human Urine material for urine albumin.

**Table S2.** The five levels for each of three factors for the DOE optimization study.

| Level | $X_1$ – Trypsin-to-Protein Mass Ratio | $X_2$ – Digestion Reaction Time | $X_3$ – Digestion Temperature |
|---|---|---|---|
| − α | 01:21.6 | 13.0 h | 28.6 °C |
| − 1 | 1:25 | 15.0 h | 32.0 °C |
| 0 | 1:30 | 18.0 h | 37.0 °C |
| 1 | 1:35 | 21.0 h | 42.0 °C |
| + α | 01:38.4 | 23.0 h | 45.4 °C |

**Table S3.** The three-factor, five-level experimental design matrix used in the DOE optimization study to determine the optimal conditions for trypsin digestion of recombinant HSA. Sample #1 to #8 represent the full factorial design matrix ($2^3$), samples #9 to #14 represent the star/axial points (± α), and samples #15 to #16 represent the center points.

| **DOE Optimization Sample Number** | **Trypsin Digestion Factor** | | | **Trypsin Digestion Factor Levels** | | |
|---|---|---|---|---|---|---|
| | **$X_1$ (Trypsin – Protein Mass Ratio)** | **$X_2$ (Trypsin Rxn Time)** | **$X_3$ (Trypsin Rxn Temp.)** | **$X_1$** | **$X_2$** | **$X_3$** |
| **1** | − 1 | − 1 | − 1 | 1:25 | 15 h | 32.0 °C |
| **2** | 1 | -1 | − 1 | 1:35 | 15 h | 32.0 °C |
| **3** | − 1 | 1 | − 1 | 1:25 | 21 h | 32.0 °C |
| **4** | 1 | 1 | − 1 | 1:35 | 21 h | 32.0 °C |
| **5** | − 1 | − 1 | 1 | 1:25 | 15 h | 42.0 °C |
| **6** | 1 | − 1 | 1 | 1:35 | 15 h | 42.0 °C |
| **7** | − 1 | 1 | 1 | 1:25 | 21 h | 42.0 °C |
| **8** | 1 | 1 | 1 | 1:35 | 21 h | 42.0 °C |
| **9** | − α (− 1.682) | 0 | 0 | 01:21.6 | 18 h | 37.0 °C |
| **10** | α (1.682) | 0 | 0 | 01:38.4 | 18 h | 37.0 °C |
| **11** | 0 | − α (− 1.682) | 0 | 1:30 | 13 h | 37.0 °C |
| **12** | 0 | α (1.682) | 0 | 1:30 | 23 h | 37.0 °C |
| **13** | 0 | 0 | − α (− 1.682) | 1:30 | 18 h | 28.6 °C |
| **14** | 0 | 0 | α (1.682) | 1:30 | 18 h | 45.4 °C |
| **15** | 0 | 0 | 0 | 1:30 | 18 h | 37.0 °C |
| **16** | 0 | 0 | 0 | 1:30 | 18 h | 37.0 °C |

**Fig S2** Graphical illustration of the three-factor, five-level experimental design matrix for the DOE optimization assessment.[2] Each point represents the factor values for one experimental sample (1 to 16), as outlined in Table 1.

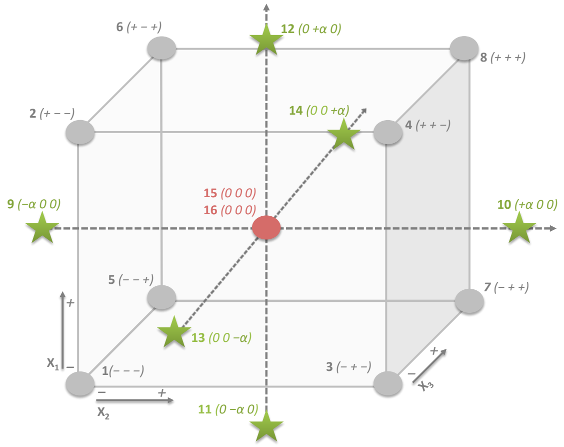

**Fig S3** Representative MRM chromatograms for the 16 samples of the DoE optimization assessment containing the 11 MRM peptides.

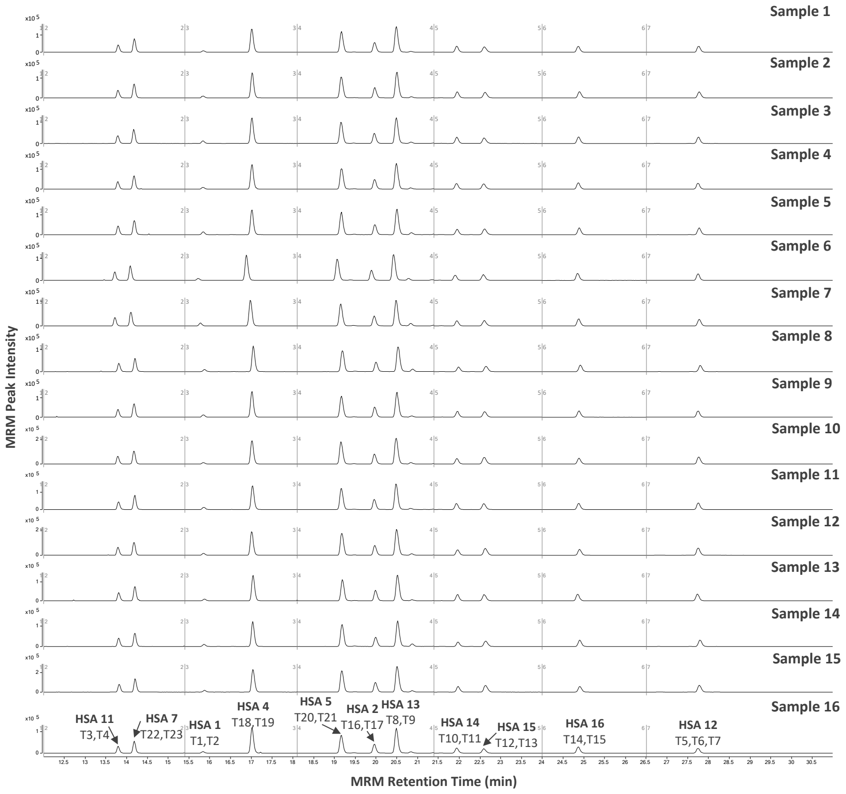


**Fig S4** Graph of peak area ratio (ratio of raw peak area for SRM 2925-to-$^{15}$N-Labeled internal standard) for each set (Set 1 – A, Set 2 – B, Set 3 – C).

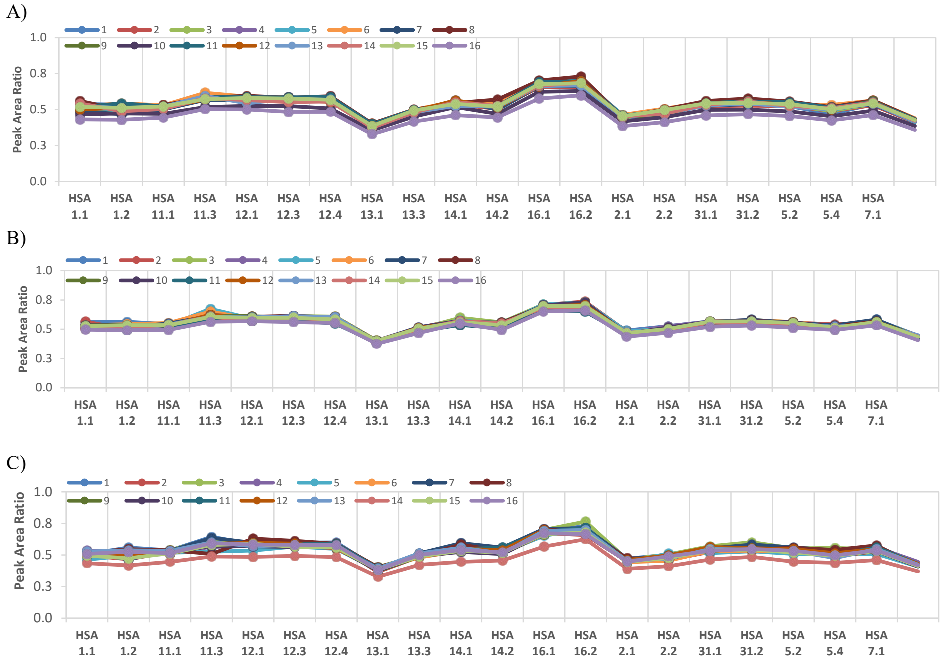

**Fig S5** Response plot of the normalized peak area mean (n=155) of the internal standard (IS,[15]N-labeled recombinant HSA) for the three sets (set 1 to 3, 16 samples per set) of the DOE optimization assessment. The error bars represent the standard error of the peak area results observed for each MRM transition across the 16 samples of the 3 sample sets.

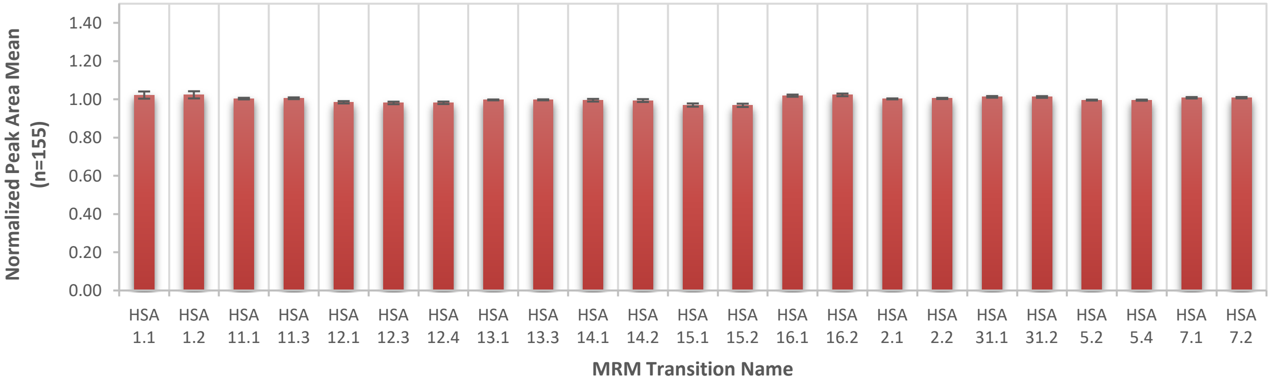

Supplement: Supplementary file 1 — Supplementary file1 (PDF 630 KB) [file 216_2023_4705_MOESM1_ESM.pdf]
